# Diet and Cardiovascular Disease Risk Among Individuals with Familial Hypercholesterolemia: Systematic Review and Meta-Analysis

**DOI:** 10.3390/nu12082436

**Published:** 2020-08-13

**Authors:** Fotios Barkas, Tzortzis Nomikos, Evangelos Liberopoulos, Demosthenes Panagiotakos

**Affiliations:** 1 Department of Internal Medicine, Faculty of Medicine, School of Health Sciences, University of Ioannina, 451 10 Ioannina, Greece; fotisbarkas@windowslive.com (F.B.); elibero@uoi.gr (E.L.); 2 Department of Nutrition & Dietetics, School of Health Science & Education, Harokopio University, 176 71 Athens, Greece; tnomikos@hua.gr

**Keywords:** diet, plant sterols, stanols, omega-3 fatty acids, familial hypercholesterolemia

## Abstract

Background: Although a cholesterol-lowering diet and the addition of plant sterols and stanols are suggested for the lipid management of children and adults with familial hypercholesterolemia, there is limited evidence evaluating such interventions in this population. Objectives: To investigate the impact of cholesterol-lowering diet and other dietary interventions on the incidence or mortality of cardiovascular disease and lipid profile of patients with familial hypercholesterolemia. Search methods: Relevant trials were identified by searching US National Library of Medicine National Institutes of Health Metabolism Trials Register and clinicaltrials.gov.gr using the following terms: diet, dietary, plant sterols, stanols, omega-3 fatty acids, fiber and familial hypercholesterolemia. Selection criteria: Randomized controlled trials evaluating the effect of cholesterol-lowering diet or other dietary interventions in children and adults with familial hypercholesterolemia were included. Data collection and analysis: Two authors independently assessed the eligibility of the included trials and their bias risk and extracted the data which was independently verified by other colleagues. Results: A total of 17 trials were finally included, with a total of 376 participants across 8 comparison groups. The included trials had either a low or unclear bias risk for most of the assessed risk parameters. Cardiovascular incidence or mortality were not evaluated in any of the included trials. Among the planned comparisons regarding patients’ lipidemic profile, a significant difference was noticed for the following comparisons and outcomes: omega-3 fatty acids reduced triglycerides (mean difference (MD): −0.27 mmol/L, 95% confidence interval (CI): −0.47 to −0.07, *p* < 0.01) when compared with placebo. A non-significant trend towards a reduction in subjects’ total cholesterol (MD: −0.34, 95% CI: −0.68 to 0, mmol/L, *p* = 0.05) and low-density lipoprotein cholesterol (MD: −0.31, 95% CI: −0.61 to 0, mmol/L, *p* = 0.05) was noticed. In comparison with cholesterol-lowering diet, the additional consumption of plant stanols decreased total cholesterol (MD: −0.62 mmol/L, 95% CI: −1.13 to −0.11, *p* = 0.02) and low-density lipoprotein cholesterol (MD: −0.58 mmol/L, 95% CI: −1.08 to −0.09, *p* = 0.02). The same was by plant sterols (MD: −0.46 mmol/L, 95% CI: −0.76 to −0.17, *p* < 0.01 for cholesterol and MD: −0.45 mmol/L, 95% CI: −0.74 to −0.16, *p* < 0.01 for low-density lipoprotein cholesterol). No heterogeneity was noticed among the studies included in these analyses. Conclusions: Available trials confirm that the addition of plant sterols or stanols has a cholesterol-lowering effect on such individuals. On the other hand, supplementation with omega-3 fatty acids effectively reduces triglycerides and might have a role in lowering the cholesterol of patients with familial hypercholesterolemia. Additional studies are needed to investigate the efficacy of cholesterol-lowering diet or the addition of soya protein and dietary fibers to a cholesterol-lowering diet in patients with familial hypercholesterolemia.

## 1. Introduction

Familial hypercholesterolemia (FH) is the most common inherited metabolic disease caused by mutations of the genes involved in low-density lipoprotein cholesterol (LDL-C) catabolism and related with premature coronary heart disease (CHD) [1,2,3]. Considering the LDL-C reduction (over 50%) needed for the prevention against the development of cardiovascular disease (CVD) in such patients, lipid-lowering drugs are considered as their primary cardiovascular (CV) prevention therapy [4,5,6]. On the other hand, dietary interventions, such as the manipulation of fat content, increasing dietary intake of soluble fiber and increasing the intake of certain dietary components (i.e., soy protein, plant sterols and stanols, omega-3 fatty acids) are recommended in patients with FH who cannot start (i.e., children) or tolerate lipid-lowering therapy (i.e., statin intolerant patients) [6,7]. Nevertheless, the majority of these interventions have not been adequately investigated in such individuals and consensus has yet to be reached on the most appropriate dietary treatment for FH [8].

The aim of this work was to assess the CV efficacy of the currently recommended cholesterol-lowering diet and other forms of dietary intervention in children and adults diagnosed with FH.

## 2. Materials and Methods 

### 2.1. Eligibility Criteria

#### 2.1.1. Types of Studies

The present meta-analysis has been conducted according to the Preferred Reporting Items for Systematic Reviews and Meta-Analyses (PRISMA) (Table A1). Published randomized controlled trials (RCTs) were included in the present meta-analysis. Trials using quasi-randomization methods were alternatively included in case of sufficient evidence showing that treatment and comparison groups were comparable in terms of clinical and nutritional status.

#### 2.1.2. Study Participants

Studies including children and adults with FH (alternative named as inherited dyslipidemia IIa) were considered eligible. Trials including patients with FH along with others not fulfilling the criteria of FH diagnosis were only included if the FH group was well defined and the results for these subjects were available. 

#### 2.1.3. Interventions

Cholesterol-lowering diet or any other dietary intervention aimed at serum total cholesterol (TC) or LDL-C reduction, for at least 3 weeks. RCTs comparing dietary treatment as a control with lipid-lowering drugs were excluded. However, we included those trials in which control and treatment groups differed only in diet. Trials comparing one form of modified dietary intake with another form of dietary intake were included in case of a head-to-head comparison.

### 2.2. Outcomes

Incidence and mortality of total CVD, CHD, stroke or peripheral arterial disease (PAD) were considered as the primary outcomes of interest in our meta-analysis. The secondary outcomes were the following: TC, triglycerides (TG), high-density lipoprotein cholesterol (HDL-C), LDL-C, very-low-density lipoprotein cholesterol (VLDL-C), apolipoprotein (apo) A-I, apoB and lipoprotein (a) (Lp(a)). 

### 2.3. Information Sources

Relevant trials were identified by searching US National Library of Medicine National Institutes of Health Metabolism Trials Register (https://www.ncbi.nlm.nih.gov/pubmed) and clinicaltrials.gov.gr (https://clinicaltrials.gov) using the following terms: diet, dietary, plant sterols, stanols, omega-3 fatty acids, fiber and familial hypercholesterolemia. RCTs included in our analysis were also scrutinized for other trials fulfilling our eligibility criteria. 

### 2.4. Data Collection and Analysis

#### 2.4.1. Selection of Studies

At the initial stage of review and each update, two authors independently selected the trials which were eligible for inclusion in the present meta-analysis.

#### 2.4.2. Data Extraction and Management

Two review authors (FB and DP) independently extracted data using an extraction form recording publication details, study population, randomization, allocation concealment, blinding, interventions and results of each trial. Any differences between them were resolved by consulting the other review authors (TN and EL). 

Due to the different dietary interventions recommended in patients with FH, the included RCTs were divided into the following comparisons:

- Dietary interventions to reduce fat content.
- Supplementation with omega-3 fatty acids compared with placebo.
- Dietary interventions modifying unsaturated fat content.
- Cholesterol-lowering diet compared with dietary interventions increasing intake of plant stanols.
- Cholesterol-lowering diet compared with dietary interventions increasing intake of plant sterols.
- Dietary interventions increasing intake of plant stanols compared with plant sterols.
- Dietary interventions modifying protein content.
- Dietary interventions increasing intake of dietary fiber.

Outcome data were grouped into those measured at up to one, three, six, twelve months and annually thereafter. In case of outcomes recorded at other time periods (i.e., 2, 4, 6, 8 weeks), the authors examined them as well. Generally, a 4-week period is adequate to investigate the effects of dietary interventions on lipids. In order to assess how these effects were maintained, analyses at longer periods were preferred. For the primary outcomes, analyzing the results of longer follow-up would have been necessary. 

In case of duplicate trials, we included the trial with the longest follow-up. 

### 2.5. Assessment of Risk of Bias in Included Studies

We assessed the bias risk (low, unclear or high) of the following parameters: (i) sequence generation, (ii) allocation concealment, (iii) blinding (of participants, personnel and outcome assessors), (iv) incomplete outcome data addressed, (v) free of selective outcome reporting and (vi) free of other bias. Overall, trials were considered as high-risk if the majority of the evaluated parameters were considered at high or unclear bias risk. Any differences between FB and DP were resolved by consultation.

### 2.6. Measurements of Treatment Effect

No data were available regarding the incidence and mortality of CVD. In case of available data for these outcomes, the odds ratio (OR) and the corresponding 95% confidence intervals (CIs) would have been estimated. 

Continuous outcomes were analyzed using the mean difference (MD) and associated 95% CIs. In case of different measurement scales, the standardized mean difference (SMD) was calculated. When only the standard error (SE) was available, it was converted to SD by being multiplied with the square root of the participant number.

### 2.7. Synthesis of Results

#### 2.7.1. Missing Data

In order to allow an intention-to-treat analysis, the authors would have sought data on the number of participants with each outcome event, by allocated treatment group, irrespective of compliance and whether or not the participant was later thought to be ineligible or otherwise excluded from treatment or follow up. 

RCTs not reporting the results of the FH subgroup have not been included in the present work. Although the authors were requested to supply this lacking data, no response was received at the time of writing this review.

#### 2.7.2. Assessment of Heterogeneity

Heterogeneity between trial results was tested using a standard chi-square test (*p* < 0.1 was considered statistically significant) and I^2^ statistic was used as a measure of heterogeneity [9]. The following ranges and descriptions were used: (i) 0–40%: might not be important, (ii) 30–60%: may represent moderate heterogeneity, (iii) 50–90%: may represent substantial heterogeneity and (iv) 75–100%: considerable heterogeneity.

### 2.8. Assessment of Reporting Biases

Publication bias was assessed with a funnel plot. Due to the lack of data on the primary outcomes, any secondary outcome reported by three or more trials was used for the funnel plot construction. Outcome reporting bias was assessed by comparing the original protocols of the included trials with the final published manuscripts. In case that the protocols were unavailable, the outcomes described in the Methods section of the final manuscripts were compared with the Results section to identify any outcomes not being reported. Finally, our clinical knowledge would help us identify any outcomes expected to be measured, but they were not reported. 

### 2.9. Subgroup Analysis and Investigation of Heterogeneity

In case of observed statistically significant heterogeneity, a random-effect meta-analysis was performed. Otherwise, a fixed-effect model was used. 

## 3. Results

### 3.1. Study Selection

**Figure 1:**
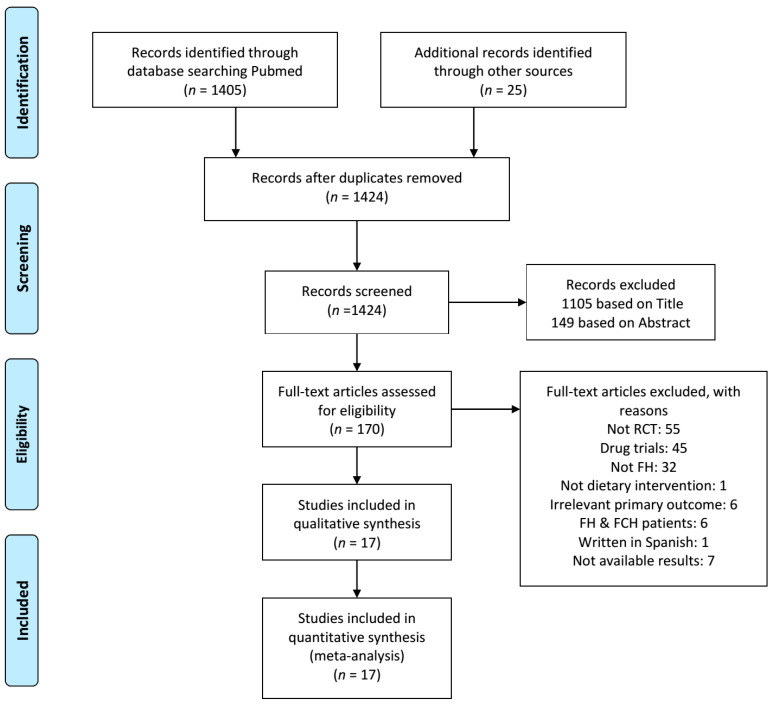
PRISMA flow diagram of study selection. FCH, familial combined hyperlipidemia; FH, familial hypercholesterolemia; RCT, randomized clinical trial.

As shown in Figure 1, of the 1430 references initially identified from the electronic and manual search studies, a total of 17 RCTs were included in the present meta-analysis. 

### 3.2. Study Characteristics

The design of the RCTs included in the present meta-analysis, along with their samples and the investigated dietary interventions are demonstrated in Table 1. The majority of the included studies were double-blind, placebo-controlled and randomized with a cross-over design [7,10,11,12,13,14,15]. Their duration ranged from 3 to 13 weeks and their samples from 10 to 62 subjects. Seven trials enrolled children fulfilling the criteria of FH [10,12,14,16,17,18,19]. Among the rest studies including adults with FH, in 8 RCTs the subjects were also treated with lipid-lowering drugs [11,13,15,20,21,22,23,24].

We report on 8 dietary interventions separately.

- Only one study evaluated the impact of cholesterol-lowering diet in adults with FH, who were treated with simvastatin [21].
- Three trials compared the effect of treatment with omega-3 fatty acids in comparison with placebo [11,13,20]. The daily supplementation of omega-3 fatty acids was 5.1 g with a ratio of eicosapentaenoic acid/ docosahexaenoic acid (EPA/DHA) of 1:1 in the oldest trial [11], whereas the treatment arm in the rest RCTs comprised of 4 g/d of EPA/DHA (46% EPA and 38% DHA) [13,20]. All of these trials included adults taking lipid-lowering therapy [11,13,20] and only one reported that its subjects adhered to cholesterol-lowering diet [11].
- Two trials evaluated the impact of modified fat on FH patients. The former compared 2 low-fat diets enriched with either monounsaturated fatty acids (MUFAs) by rapeseed oil or polyunsaturated fatty acids (PUFAs) by sunflower oil in children with FH [19]. The second trial assigned its subjects to 2 cholesterol-lowering diets differing with regard to polyunsaturated:saturated values (2.0 and 1.3, respectively) [25].
- Two RCTs investigated the dietary interventions increasing the intake of plant stanols. The first study compared the addition of 3 g sitostanol dissolved in margarine to cholesterol-lowering diet with placebo in children with FH [16]. The second one evaluated the addition of 2 g plant stanols to cholesterol-lowering diet in a fortified yogurt in comparison with placebo in children with FH [14].
- Four trials evaluated the addition of plant sterols to cholesterol-lowering diet compared with placebo in FH patients [10,12,15,22]. Plants sterols were administered in a fortified margarine spread at a dose ranging 1.6-2.5 g/d. Two of the trials included children with FH [10,12] and the rest studies included FH adults receiving lipid-lowering drugs [15,22]. One trial compared the addition of 2 g/d plant stanols with 2 g/d plant sterols in FH adults who adhered to cholesterol-lowering diet and were on lipid-lowering therapy [23].
- Three RCTs evaluated dietary interventions modifying the protein content of the diet in FH patients [17,18,26]. Two of these trials manipulated protein content by increasing the consumption of soy protein [17,18]. The former compared 2 different cholesterol-lowering diets with high-protein content in which 35% of the protein was consumed as dairy source, either from soy beverage or cow milk [18]. The latter RCT investigated the addition of soy-protein to a diet high in unsaturated and low in saturated fats compared with placebo [17]. Both of these RCTs referred to children with FH [17,18]. The third trial investigated the increase in protein intake on top of a cholesterol-lowering diet in FH adults [26].
- Only one trial investigated the impact of dietary fibers on FH adults [24]. In this RCT, treatment with guar gum and bezafibrate was compared with bezafibrate alone [24]. The authors did not report whether their subjects adhered to cholesterol-lowering diet or not [24].

### 3.3. Bias Risk within Studies

**Figure 2:**
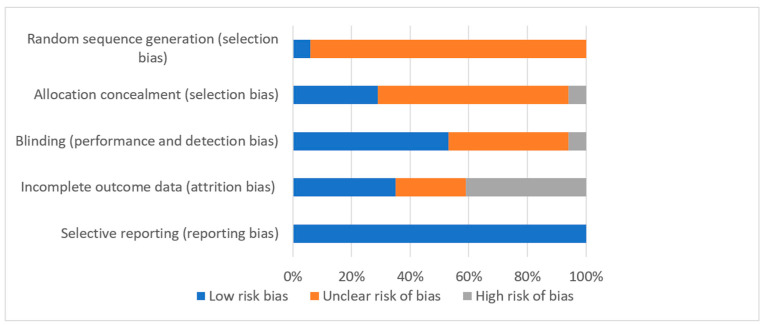
Bias risk graph. Judgments about each risk of bias item are presented as percentages across all included studies.

The included trials had either a low or unclear bias risk for most of the parameters used for risk assessment (Figure 2).

#### 3.3.1. Allocation

Only one trial reported adequately on the randomization sequence; they stated that computer-generated random numbers were used for the assignment to either test or the control group with equal probability [15]. Reports on the generation of the randomization sequence were unclear in the remaining 16 trials [10,11,12,13,14,16,17,18,19,20,21,22,23,24,25,26]. 

Concealment of allocation was adequate in 5 trials where the methods used for allocation concealment were extensively described [12,13,14,15,19]. One trial was considered to be at high bias risk due its open-label design [20]. On the other hand, data regarding allocation concealment was unclear in the rest RCTs [10,11,16,17,18,21,22,23,24,25,26].

#### 3.3.2. Blinding

Nine RCTs were reported as being double-blinded [10,11,12,13,14,15,16,19,23]. One RCT was open-label [20], whereas the rest trials did not provide any information regarding blinding [17,18,21,22,24,25,26].

#### 3.3.3. Incomplete Outcome Data

It was unclear whether an intention-to-treat analysis was carried out in one of the trials, giving thus an unclear risk of bias [21]. Intention-to-treat analysis was adequate in 6 RCTs giving a low risk of bias [7,12,14,15,23,26]. In 7 RCTs participants were withdrawn and not included in the final analysis; consequently intention-to-treat analysis was not applied [10,11,13,18,19,20,22]. One trial undertook a per protocol analysis [17] and no sample attrition was performed in two RCTs [24,25].

#### 3.3.4. Selective Reporting

No selective reporting was noted in the included RCTs. 

### 3.4. Effects of Interventions

Only 11 RCTs presented data in such way that the preferred method of analysis could be conducted [10,11,12,13,14,15,16,17,18,20,22]. However, these trials did not provide data for all of the assessed outcomes. Furthermore, no RCT reported on the incidence or mortality of total CVD, CHD, stroke and PAD. 

#### 3.4.1. Dietary Interventions Reducing Fat Intake 

Low-fat diet had no impact on subjects’ TC (MD: −0.40 mmol/L, 95% CI: −0.95 to 0.15), TG (MD: 0.06 mmol/L, 95% CI: −0.43 to 0.55), HDL-C (MD: −0.11 mmol/L, 95% CI: −0.34 to 0.12), LDL-C (MD: −0.27 mmol/L, 95% CI: −0.79 to 0.25) and VLDL-C (MD: 0.01 mmol/L, 95% CI: −0.24 to 0.26), when compared with a higher-fat diet (Table S1) [21].

#### 3.4.2. Supplementation with Omega-3 Fatty Acids Compared with Placebo

The lipid profile of subjects participating in the RCTs evaluating the administration of omega-3 fatty acids are demonstrated in Table S2 [11,13,20]. 

**Figure 3:**
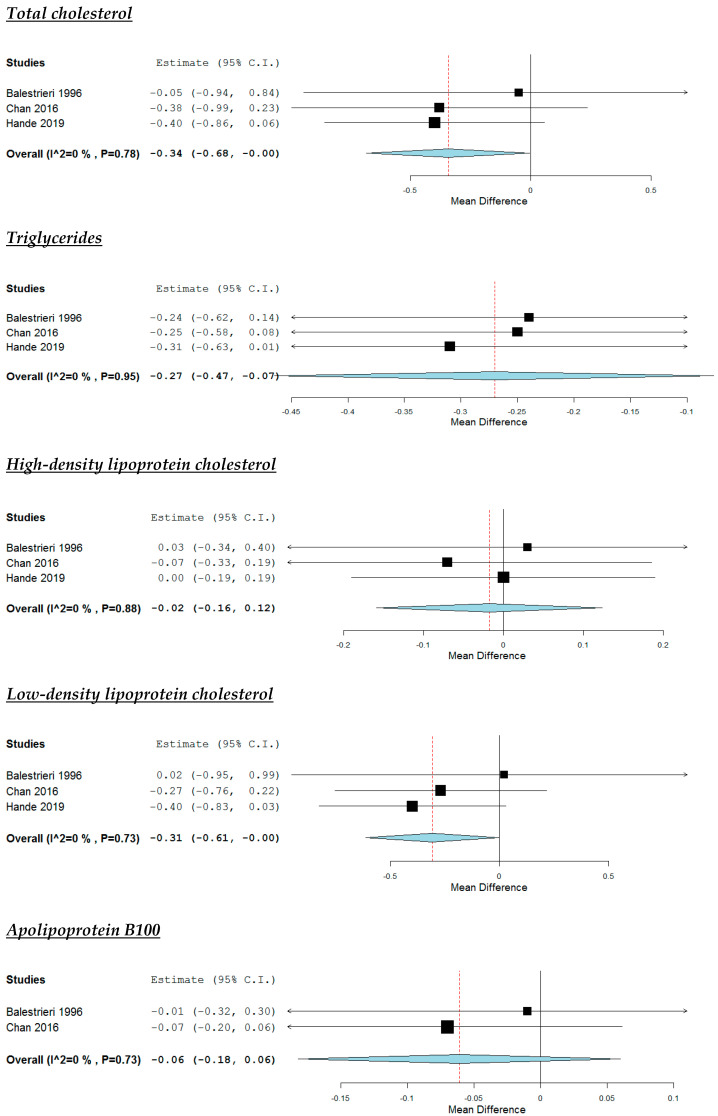
Effect of supplementation with omega-3 fatty acids compared with placebo.

According to the pooled analysis (Figure 3), the supplementation with omega-3 fatty acids decreased study participants’ TG (MD: −0.27 mmol/L, 95% CI: −0.47 to −0.07, *p* <0.01), but had no impact on their HDL-C (MD: −0.02 mmol/L, 95% CI: −0.16 to 0.12) and apoB100 (MD: −0.06 g/L, 95% CI: −0.18 to 0.06). A non-significant trend towards a reduction in subjects’ TC (MD: −0.34 mmol/L, 95% CI: −0.68 to 0, *p* = 0.05) and LDL-C (MD: −0.31 mmol/L, 95% CI: −0.61 to 0, *p* = 0.05) was noticed (Figure 1). No significant heterogeneity was noticed across studies (Figure 3).

Individual studies showed that omega-3 fatty acids decreased subjects’ VLDL-C (MD: −0.20 mmol/L, 95% CI: −0.23 to −0.16, *p* < 0.05) [20], but no effect was noticed regarding their apoA-I (MD: 0.02 g/L, 95% CI: −0.31 to 0.35) and Lp(a) (MD: −0.02 g/L, 95% CI: −0.31 to 0.27) (Table S2) [11].

#### 3.4.3. Dietary Interventions Modifying Unsaturated Fat Content

##### Low-Fat Diet Regimes Enriched with either Monounsaturated Fatty Acids or Polyunsaturated Fatty Acids

The trial comparing two low-fat diet regimes enriched with either MUFAs or PUFAs showed no difference between 2 groups regarding subjects’ TC (MD: −0.73 mmol/L, 95% CI: −1.69 to 0.23), TG (MD: −0.03 mmol/L, 95% CI: −0.53 to 0.47), HDL-C (MD: 0.10 mmol/L, 95% CI: −0.19 to 0.39), LDL-C (MD: −0.84 mmol/L, 95% CI: −1.90 to −0.22), apoA-I (MD: −0.01 g/L, 95% CI: −0.25 to 0.23) and apoB100 (MD: −0.09 g/L, 95% CI: −0.36 to 0.18) (Table S3) [19].

##### Cholesterol-Lowering Diets Differing with Regard to Polyunsaturated:Saturated Values

One study showed that that increasing the PUFAs:saturated fat value of lipid-lowering diets from 1.3 to 2.0 did not offer a great advantage with regard to reduction in subjects’ TC (0.03 ± 0.64 mmol/L), TG (−0.01 ± 0.23 mmol/L), HDL-C (0 ± 0.13 mmol/L), LDL-C (0.02 ± 0.06 mmol/L) and VLDL-C (0.09 ± 0.13 mmol/L) [25].

#### 3.4.4. Cholesterol-Lowering Diet Compared with Dietary Interventions Increasing Intake of Plant Stanols

The lipid profile of subjects participating in the RCTs evaluating the dietary interventions increasing the intake of plant stanols are demonstrated in Table S4 [14,16]. 

**Figure 4:**
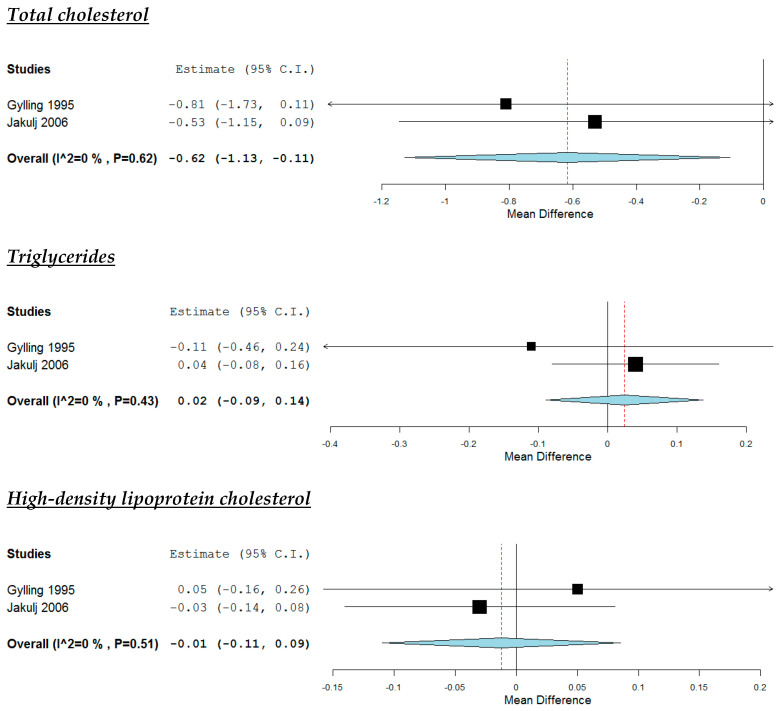

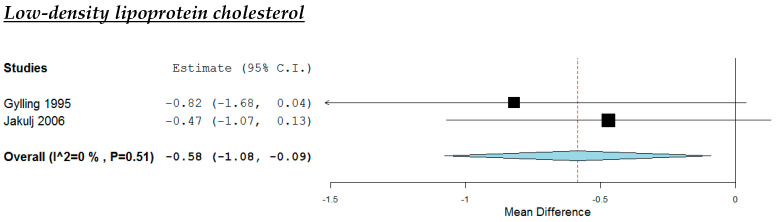
Effect of increased intake of plant stanols compared with placebo.

According to the pooled analysis (Figure 4), the increased intake of plant stanols reduced study participants’ TC (MD: −0.62 mmol/L, 95% CI: −1.13 to −0.11, *p* = 0.02) and LDL-C (MD: −0.58 mmol/L, 95% CI: −1.08 to −0.09, *p* = 0.02), but they had no impact on their TG (MD: −0.02 mmol/L, 95% CI: −0.09 to 0.14) and HDL-C (MD: −0.01 mmol/L, 95% CI: −0.11 to 0.09). No significant heterogeneity was noticed across studies (Figure 4).

One study showed that plant stanols had no impact on subjects’ VLDL-C (MD: −0.08 mmol/L, 95% CI: −0.26 to 0.10) (Table S4) [16]. 

#### 3.4.5. Cholesterol-Lowering Diet Compared with Dietary Interventions Increasing Intake of Plant Sterols

The lipid profile of subjects participating in the RCTs evaluating the dietary interventions increasing the intake of plant sterols are demonstrated in Table S5 [10,12,15,22]. 

**Figure 5:**
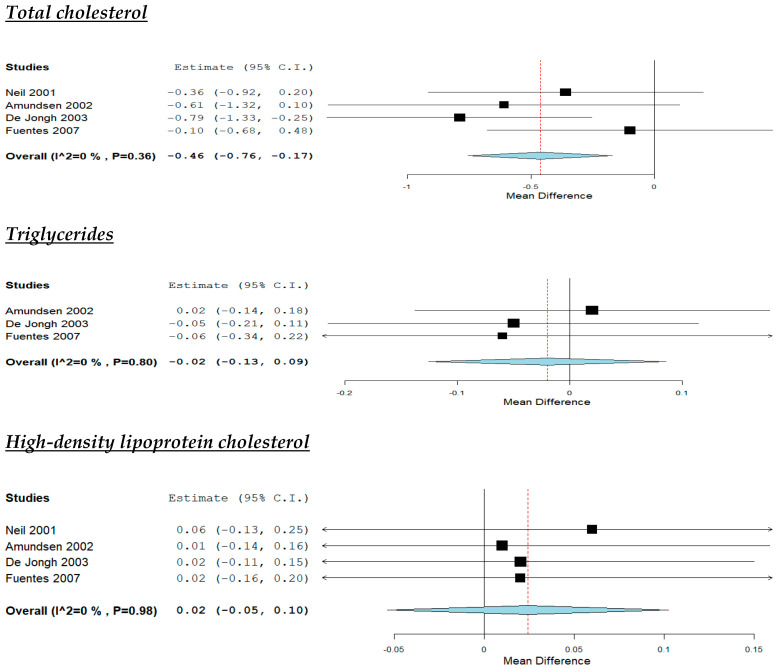

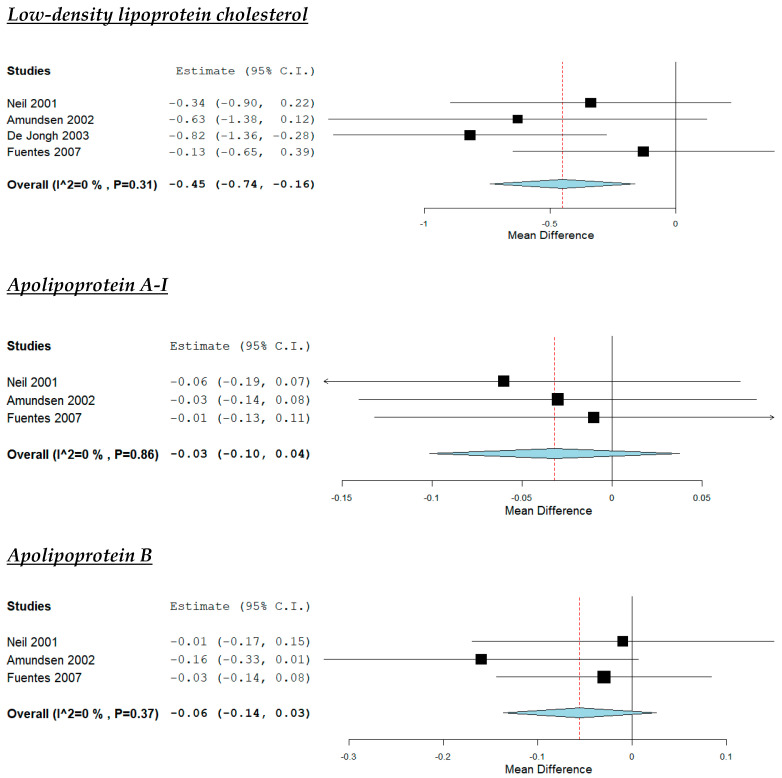
Effect of increased intake of plant sterols compared with placebo.

According to the pooled analysis (Figure 5), the increased intake of plant stanols reduced study participants’ TC (MD: −0.46 mmol/L, 95% CI: −0.76 to −0.17, *p* < 0.01) and LDL-C (MD: −0.45 mmol/L, 95% CI: −0.74 to −0.16, *p* < 0.01). On the other hand, no effect was noticed regarding their TG (MD: −0.02 mmol/L, 95% CI: −0.13 to 0.09, HDL-C (MD: 0.02 mmol/L, 95% CI: −0.05 to 0.1,), apoA-I (MD: −0.03 g/L, 95% CI: −0.10 to 0.04) and apoB (MD: −0.06 g/L, 95% CI: −0.14 to 0.03) No significant heterogeneity was noticed across studies (Figure 5).

One study showed no impact on VLDL-C (MD: −0.08 mmol/L, 95% CI: −0.26 to 0.10) (Table S5) [15].

#### 3.4.6. Dietary Interventions Increasing Intake of Plant Stanols Compared with Plant Sterols

There was no difference between the addition of 2 g/d plant stanols and 2 g/d plant sterols in FH adults who adhered to cholesterol-lowering diet regarding their TC (MD: −0.06 mmol/L, 95% CI: −0.66 to 0.54), TG (MD: 0.11 mmol/L, 95% CI: −0.18 to 0.40), HDL-C (MD: −0.05 mmol/L, 95% CI: −0.16 to 0.06) and LDL-C (MD: −0.05 mmol/L, 95% CI: −0.56 to 0.46) (Table S6) [23]. 

#### 3.4.7. Dietary Interventions Modifying Protein Content

##### Soy Protein as a Form of Dietary Intervention Compared to Another Form or no Intervention

The lipid profile of subjects participating in the RCTs evaluating the dietary interventions increasing soy protein intake is demonstrated in Table S7 [17,18].

**Figure 6:**
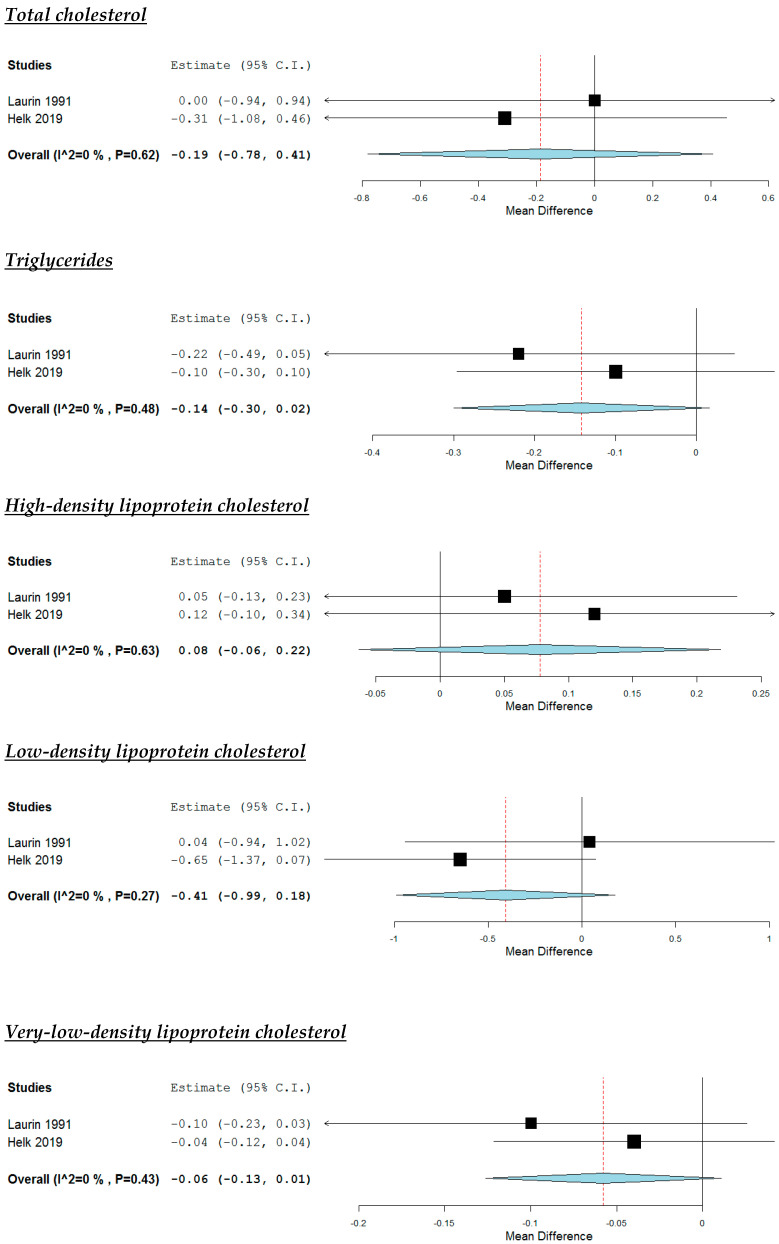

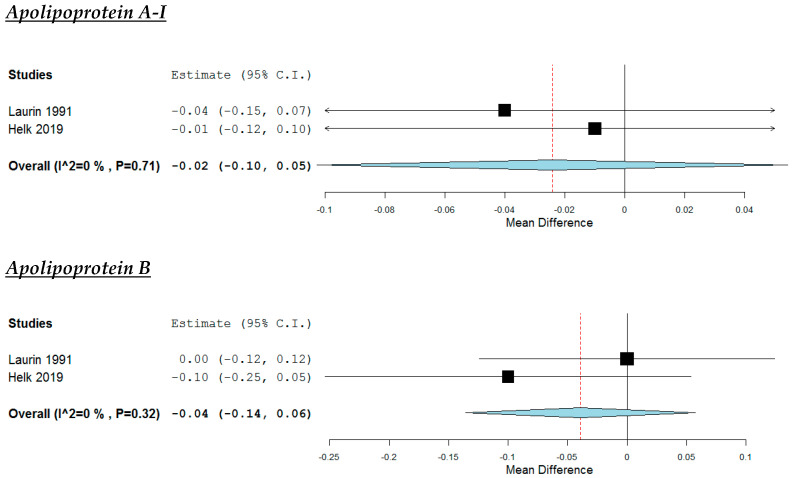
Effect of increased intake of soy protein compared with control group.

According to the pooled analysis (Figure 6), the dietary interventions increasing soy intake had no impact on study participants’ TC (MD: −0.19 mmol/L, 95% CI: −0.78 to 0.41), TG (MD: −0.14 mmol/L, 95% CI: −0.30 to 0.02), HDL-C (MD: 0.08 mmol/L, 95% CI: −0.06 to 0.22L), LDL-C (MD: −0.41 mmol/L, 95% CI: −0.99 to 0.18), VLDL-C (MD: −0.06 mmol/L, 95% CI: −0.13 to 0.01), apoA-I (MD: −0.02 g/L, 95% CI: −0.10 to 0.05) and apoB (MD: −0.04 g/L, 95% CI: −0.14 to 0.06). No significant heterogeneity was noticed across studies, apart from the analysis concerning LDL-C (Figure 6).

One study showed that soy had no impact on subjects’ Lp(a) (MD: −0.29 g/L, 95% CI: −0.65 to 0.07) (Table S7) [17].

##### Dietary Intervention to Increase Protein Intake

The dietary interventions increasing protein intake reduced subjects’ TG (MD: −0.70 mmol/L, 95% CI: −1.32 to −0.08, *p* < 0.05) and LDL-C (MD: −0.30 mmol/L, 95% CI: −0.85 to −0.25, *p* < 0.05), but had no impact on their TC (MD: −0.40 mmol/L, 95% CI: −1.23 to 0.43), VLDL-C (MD: −0.17 mmol/L, 95% CI: −0.44 to 0.10) and HDL-C (MD: 0.08 mmol/L, 95% CI: −0.14 to 0.10) (Table S8) [26].

#### 3.4.8. Dietary Interventions to Increase Intake of Dietary Fiber

The dietary interventions increasing dietary fiber intake decreased subjects’ LDL-C (MD: −1.83 mmol/L, 95% CI: −3.32 to −0.34, *p* < 0.05) and apoB (MD: −0.50 g/L, 95% CI: −0.65 to −0.35, *p* < 0.05). On the other hand, guar had no impact on their TC (MD: −0.57 mmol/L, 95% CI: −2.08 to 0.94), TG (MD: 0.41 mmol/L, 95% CI: −0.12 to 0.94), HDL-C (MD: −0.18 mmol/L, 95% CI: −0.47 to 0.11) and apoA-I (MD: 0.04 g/L, 95% CI: −0.05 to 0.13) (Table S9) [24].

## 4. Discussion

The present meta-analysis included 17 RCTs evaluating the impact of different dietary interventions on lipid levels of children and adults diagnosed with FH. No RCT investigating the impact of dietary interventions on CVD incidence or mortality was found. According to our pooled analyses, increased intake of plants sterols and stanols by fortified foods reduced TC and LDL-C in such individuals. Although a non-significant trend towards a reduction in TC and LDL-C was noticed, supplementation with omega-3 fatty acids resulted in TG decrease in this population.

FH is the most commonly inherited metabolic disease and associated with premature CVD, if left untreated [3,4,5,27,28]. Considering LDL-C reduction (over 50%) needed for the prevention against CVD development in FH patients, lipid-lowering drugs are the primary CV prevention therapy in such individuals [4,5,6]. Statins remain the cornerstone treatment and current guidelines recommend treating FH adults and FH children >8 years old with maximally tolerated doses of high-intensity statins, which are capable of lowering LDL-C ≥ 50% [4,5,6,29,30]. Ezetimibe and PCSK9 inhibitors are additional therapeutic options reducing LDL-C by 20–60%, in case that the patients are statin intolerant or do not achieve optimal LDL-C levels; of note, the latter has not been approved yet in children [4,5,6,29,30]. Novel lipid-lowering drugs, such as inclisiran, angiopoietin-like 3 protein, bempedoic acid and gemcabene are a few therapeutic options currently investigated for the future management of such individuals [5]. Despite the available effective lipid-lowering drugs, a considerable proportion of patients diagnosed with FH remain suboptimally treated in clinical practice [3,31]. In addition, there are patients diagnosed with FH who cannot be treated with lipid-lowering drugs, such as statin-intolerant or pregnant patients and children aged <8 years old [6]. In this context, dietary interventions including diet modification or dietary supplements might be helpful, if not necessary, in FH individuals. Indeed, current guidelines propose manipulating dietary fat, increasing fiber intake or certain dietary components for the management of patients with dyslipidemia, whereas phytosterols are recommended in hypercholesterolemic patients at low CV risk not qualifying for pharmacotherapy or as an adjunct to lipid-lowering therapy in those at high CV risk and in patients who cannot start (i.e., children or pregnant women with FH) or tolerate lipid-lowering therapy (i.e., statin intolerant patients) [6,7]. However, the majority of these interventions have not been adequately investigated in patients with FH.

Although cholesterol-lowering diet is the primary dietary suggestion in patients diagnosed with FH, only one study including FH adults has compared low-fat/low-cholesterol diet with a diet of higher content in fat and cholesterol and showed no difference between 2 interventions [21]. However, it has to be noticed that no data were available regarding the fat quality in subjects’ diet [21]. Therefore, considering the fact that reduction of total fat intake is not so important as the modification of fat quality (i.e., replacement of dietary trans fatty acids with PUFAs) in CV prevention and cholesterol reduction [32,33], the results of Chisholm et al. are insufficient to reach any conclusion on the efficacy of cholesterol-lowering diet in FH patients. Increasing evidence has shown that FH individuals developing CHD exhibit risk factors associated with metabolic syndrome and insulin resistance, such as elevated TG, fasting blood glucose, obesity and hypertension or increased susceptibility to coagulopathy [34]. In this context, low-carb diet has been alternatively proposed over low-fat diet in FH patients with an insulin-resistant phenotype or increased thrombotic risk and the conduction of future trials assessing the effects of a low-carb diet on such individuals has been recently suggested [34]. As expected, the FH subjects of the included RCTs in the present analysis did not have increased TG or low HDL-C (Supplementary Materials), but the majority of the adult study participants were overweight (Table 1). Therefore, irrespectively of which diet will prove superior regarding the lipid management and CV prevention in FH patients, any dietary intervention reducing dietary intake is effective in weight reduction or control and should be recommended in such individuals, especially in those with overweight or obesity [35,36]. More importantly, diet should be a part of a holistic therapeutic plan ensuring patients’ compliance and aiming at the improvement of CVD-related lifestyle factors, such as smoking, physical activity and body mass index control [37]. 

Similar to previous meta-analyses including dyslipidemic patients not fulfilling the criteria of FH [38], ours demonstrated that supplementation with omega-3 fatty acids significantly reduced TG, but had no impact on HDL-C levels of FH individuals. On the other hand, our results showing a non-significant trend towards a reduction in TC and LDL-C support the conflicting evidence regarding the impact of omega-3 fatty acids on cholesterol [38,39,40]. In this context, additional studies are needed to evaluate different quantity of EPA/DHA or quality of omega-3 fatty acids on FH patients’ cholesterol indices. Indeed, REDUCE-IT trial which assigned its subjects to icosapent ethyl, a highly purified eicosapentaenoic acid ethyl ester or placebo showed that the former was associated with a significant non-HDL-C and apoB reduction [41]. Considering the fact that these are more accurate markers of the total atherogenic lipoproteins accounting for residual CVD risk than LDL-C [42], along with the fact that the insulin-resistant FH individuals are more prone to CVD development [34], omega-3 fatty acids could be beneficial in this subset of patients. 

One trial comparing 2 cholesterol-lowering diets enriched with either MUFAs or PUFAs in FH patients did not confirm available evidence supporting that PUFAs may have a greater impact on LDL-C reduction than MUFAs [19,43]. Similarly, the replacement of saturated fat with PUFAs had no impact on FH patients’ lipid profile in another study [25]. Nevertheless, the controversial results of these studies should be taken into account after considering the lack of data on their subjects’ fat quality and the limitations regarding their small sample and design. 

Undoubtedly, plant sterols and stanols are effective lipid-lowering dietary interventions and suggested by current guidelines for the management of dyslipidemias [44,45,46]. Not only our results confirmed previous evidence, but also showed that the cholesterol-lowering benefit of phytosterols seems greater in FH individuals; the average LDL-C reduction was 0.45–0.58 mmol/L in our analyses, whereas the corresponding reduction was 0.34 mmol/L in another one including RCTs with dyslipidemic individuals [44]. On the other hand, our results did not confirm available evidence supporting that phytosterols may also lower TG in normotriglyceridemic individuals [45,46].

Only one study has performed head-to-head comparisons between phytosterols in FH patients and showed no difference between 2 groups [23]. According to our results, a greater LDL-C reduction was noticed in the case of plant stanols rather than plant sterols (0.58 vs. 0.45 mmol/L). Despite not being significant, a similar trend was demonstrated by another meta-analysis including studies with hypercholesterolemic patients (MD: −0.13 mmol/L, 95% CI: −0.38 to 0.12, for the comparison between plant stanols and sterols) [44]. 

A regression analysis including a total of 312,175 participants from 49 RCTs with 39,645 major vascular events showed that bot statin and non-statin therapies (diet, bile acid sequestrants, ileal bypass and ezetimibe) were associated with similar risk ratios (RR) of major vascular events per 1-mmol LDL-C reduction (RR: 0.77, 95% CI: 0.71−0.84 for statins and RR: 0.75, 95% CI: 0.66–0.86 for non-statin therapies) [47]. Considering the linear association between LDL-C and CVD risk reduction noticed in that analysis, the addition of plant sterols or stanols lowering LDL-C by ~0.50 mmol/L in the FH patients would reduce their CVD risk by ~12.5% according to our results [47]. In addition, it has been proposed that phytosterols are cost-effective in reducing lifetime LDL-C burden in FH children [48]. Therefore, despite their small cholesterol-lowering effect, phytosterols could attenuate the efficacy of lipid-lowering drugs on CVD prevention in such individuals.

Our pooled analysis of 2 RCTs did not confirm the beneficial effect of increased soy consumption on cholesterol reduction [49]. However, it has to be noticed that apart from the limited number of the included RCTs in the analysis and their small sample, their control groups differed. The former compared 2 cholesterol-lowering/high-protein diets with increased intake of either soy protein or cow milk [18] and the latter compared a soy-enriched fat modified diet with a fat modified diet [17]. On the other hand, a small RCT demonstrated that increased protein intake decreased FH patients’ LDL-C and TG [26]. Of note, no data were available regarding subjects’ protein food sources. Therefore, future studies are needed in order to confirm the cholesterol-lowering effect of increased intake of soy protein in individuals diagnosed with FH. 

Finally, the only RCT evaluating the impact of increased guar intake in FH patients has confirmed available evidence supporting the beneficial effect of dietary fiber on lipids [50,51]. 

Our results should be considered under certain limitations. First, only a few RCTs have investigated the impact of dietary interventions in patients with FH. Not only their samples were small, but also, they were short-term. In addition, the criteria for FH diagnosis was not defined in all studies and only almost half RCTs included patients taking lipid-lowering therapy. Finally, publication bias cannot be ruled out; there was no adequate data to assess selection, performance and detecting bias. However, a high-risk attrition bias was noticed. On the other hand, the present meta-analysis is the most recent to amplify the limited bibliography reporting on the impact of diet on FH patients [52,53,54]. Malhotra et al. were the last to perform a similar meta-analysis to ours in 2014 and confirm only the lipid-lowering effect of plant sterols on FH individuals [54]. In contrast to them, we included 7 additional RCTs in the present meta-analysis. Of note, a few methodological issues should be considered in the previous meta-analysis by Malhotra et al. Two RCTs included in their pooled analyses did not report separately on the subgroup of FH patients [55,56]. In addition, their pooled analysis evaluating the dietary interventions increasing the intake of plant stanols included 2 RCTs; the former assigned their participants to plant stanols and placebo, but the latter assigned their subjects to plant stanols and plant sterols [16,57]. Finally, their pooled analysis evaluating protein intake included 2 trials with different dietary interventions. As already mentioned, Laurin et al. compared 2 low-fat/high-protein diets enriched by either soy protein or cow milk and Wolfe et al. compared a high- with a low-protein diet [18,26]. Therefore, our meta-analysis provides valuable data regarding the role of dietary interventions in CV prevention in FH patients. The addition of plant sterols and stanols to cholesterol-lowering diet, along with omega-3 fatty acids supplementation undoubtedly reduce cholesterol and TG in such individuals. However, future trials are needed to confirm the benefit of cholesterol-lowering diet and soy intake in this population. Last but not least, long RCTs are needed to elucidate the impact of such interventions on CVD incidence and mortality.

## 5. Conclusions

No robust conclusions can be reached about the impact of a cholesterol-lowering diet or any of the other dietary interventions proposed for FH patients on CVD incidence or mortality. Available RCTs confirm that the addition of plant sterols or stanols to low-fat diet has a cholesterol-lowering effect on such individuals. Considering their beneficial effect on lifetime LDL-C burden, phytosterols should be recommended in FH patients, especially in the children. On the other hand, supplementation with omega-3 fatty acids effectively reduces TG and might have a role in those exhibiting an insulin-resistant phenotype. Additional RCTs are needed to investigate the effectiveness of cholesterol-lowering diet and the addition of soy protein and dietary fibers to a cholesterol-lowering diet in patients with FH. Until then, physicians should keep in mind that diet aiming at weight reduction or control should be an integral part of a holistic approach aiming at the improvement of lifestyle CV risk factors. 

## Figures and Tables

**Table 1:** Characteristics of the included trials.

Trial	Study Design (Duration)	Participants	Interventions
Amundsen 2002 [10]	Double-blind, placebo-controlled randomized, cross-over (8w)	41 children with FH (aged 10.5 ± 1.7 yrs old, mean BMI 18.9 kg/m^2^)	Low-fat/low-cholesterol diet and 1.60 ± 0.13 g plant sterols in a fortified spread (18.2 ± 1.5 g/d) vs. low-fat/low-cholesterol diet and placebo
Balestrieri 1996 [11]	Double-blind, randomized, cross-over (4w)	16 adults with FH treated with simvastatin (aged 45.2 ± 15 yrs old)	Cholesterol-lowering diet and 6 g/d fish oil ethyl ester vs. cholesterol-lowering diet and placebo (olive oil)
Chan 2016 [20]	Open-label, placebo-controlled randomized, cross-over (8w)	22 adults with FH taking lipid-lowering therapy (aged 53.3 ± 3 yrs old, mean BMI 27 ± 1.4 kg/m^2^)	4 g/d omega-3 fatty acid ethyl ester (46% eicosapentaenoic acid and 38% docosahexaenoic acid) vs. placebo
Chisholm 1994 [21]	Randomized, cross-over (8w)	19 adults with FH treated with simvastatin (aged 51 ± 10 yrs old, mean BMI 28.7 ± 1.2 kg/m^2^)	Low-fat/low-cholesterol diet vs. a higher-fat/higher-cholesterol diet
De Jongh 2003 [12]	Double-blind, placebo-controlled randomized, cross-over (4w)	41 children with FH (aged 9.2 ± 1.6 yrs old, mean BMI 17.7 kg/m^2^) and 20 controls (aged 8.2 ± 2.2 yrs old, mean BMI 17.5 kg/m^2^)	Low-fat/low-cholesterol diet and 2.3 g plant sterols in a fortified spread (15 g/d) vs. low-fat/low-cholesterol diet and placebo
Fuentes 2008 [22]	Randomized, cross-over (4w)	30 adults with FH taking lipid-lowering therapy (aged 42 ± 18 yrs old, mean BMI 26.5 ± 3.7 kg/m^2^)	4 low-fat diets with different content of cholesterol (<150 or 300 mg/d) and sitosterol (<1 or 2 g/d)
Gustafsson 1983 [25]	Randomized, cross-over (3w)	20 hyperlipoproteinemic adults: 6 with type IIa (aged 30–60 yrs old), 8 with type IIb (aged 41–65 yrs old) and 6 with type IV hyperlipoproteinemia (aged 51–66 yrs old)	2 low-cholesterol diets differing in polyunsaturated:saturated fat ratio (2.0 vs. 1.3)
Gylling 1995 [16]	Double-blind, placebo-controlled randomized, cross-over (6w)	14 children with heterozygous FH (aged 9.1 ± 1.1 yrs old, mean BMI 17.7 ± 0.9 kg/m^2^)	Low-fat/low-cholesterol diet and 3 g sitostanol ester dissolved in rapeseed oil margarine vs. low-fat/low-cholesterol diet and placebo
Hande 2019 [13]	Double-blind, placebo-controlled randomized, cross-over (3m)	34 patients with FH on lipid-lowering treatment (aged 46.6 (18–71) yrs old, mean BMI 27.6 ± 5 kg/m^2^)	4 g/d omega-3 fatty acids in a 1000 mg capsule consisting of 460 mg of eicosapentaenoic acid and 380 mg of docosahexaenoic acid (administered twice a day) vs. placebo (capsules with olive oil)
Helk 2019 [17]	Placebo-controlled randomized (13w)	26 children with FH (aged 8.7 ± 3.8 yrs old, mean BMI 16.3 ± 3.1 kg/m^2^)	Diet high in unsaturated fats, low in saturated fats and enriched with soy-protein vs. diet high in unsaturated fats and low in saturated fats
Jakulj 2006 [14]	Double-blind, placebo-controlled randomized, cross-over (4w)	42 children with FH (aged 9.8 ± 1.5 yrs old, mean BMI 17.7 ± 2.8 kg/m^2^)	Low-fat/low-cholesterol diet and 2 g plant stanols in a low-fat fortified yogurt (500 mL/d) vs. low-fat/low-cholesterol diet and placebo
Ketomaki 2005 [23]	Double-blind randomized, cross-over (4w)	18 adults with FH taking lipid-lowering therapy (aged 48 ± 2 yrs old)	Low-fat diet and 2 g plant stanols (25 g spread/d) vs. low-fat diet and 2 g plant sterols (25 g spread/d)
Laurin 1991 [18]	Randomized, cross-over (4w)	10 children with FH (aged 8 ± 1 yrs old, mean BMI 16.7 ± 0.9 kg/m^2^)	2 different Low-fat/low-cholesterol/high-protein diets: about one-third (35%) of the protein energy was consumed as a dairy source, either from cow milk or a soy beverage
Negele 2015 [19]	Double-blind, randomized pilot trial (13w)	21 children with FH (aged 11.1 ± 3.4 yrs old, mean BMI: 19.1 ± 3.5 kg/m^2^)	Low-fat/low-cholesterol diet and monounsaturated fatty acids by rapeseed oil vs. low-fat/low-cholesterol diet and polyunsaturated fatty acids by sunflower oil
Neil 2001 [15]	Double-blind, placebo-controlled randomized, cross-over (8w)	62 adults with heterozygous FH (30 were statin-treated) (aged 51.6 (33.3–62.3) yrs old, mean BMI 25.9 ± 3.5 kg/m^2^)	Low-cholesterol diet and 2.5 g plant sterols in a fortified spread (25 g/d) vs. low-cholesterol diet and placebo
Wirth 1982 [24]	Randomized cross-over (2m)	12 adults with FH treated with fibrate (aged 51.7 (31–60) yrs old, mean BMI 26.6 kg/m^2^)	Bezafibrate vs. bezafibrate and 5.2 g guar
Wolfe 1992 [26]	Randomized, cross-over (4-5w)	10 adults with familial hypercholesterolemia (2 of those had possibly FCH) (aged 50 ± 5 yrs old, with mean BMI 24.4 kg/m^2^)	Low-fat/low-cholesterol/high-protein (23%) diet vs. low-fat/low-cholesterol/low-protein (11%) diet

BMI, body mass index; d, day; FCH, familial combined hyperlipidemia; FH, familial hypercholesterolemia; m, months; w, weeks; yrs, years.

## Supplementary Materials

The following are available online at https://www.mdpi.com/2072-6643/12/8/2436/s1, Table S1. Lipid profile of subjects assigned to low-fat/low-cholesterol diet and high-fat/high-cholesterol diet. Table S2. Lipid profile of subjects assigned to omega-3 fatty acids and placebo. Table S3. Lipid profile of subjects assigned to low-fat diet regimes enriched with either monounsaturated fatty acids or polyunsaturated fatty acids. Table S4. Lipid profile of subjects assigned to plant stanols and placebo. Table S5. Lipid profile of subjects assigned to plant sterols and placebo. Table S6. Lipid profile of subjects assigned to plant stanols and plant sterols. Table S7. Lipid profile of subjects assigned to soy protein and control group. Table S8. Lipid profile of subjects assigned to increased and low protein intake. Table S9. Lipid profile of subjects assigned to bezafibrate plus guar and bezafibrate alone.

## Appendix A

**Table A1:** PRISMA Checklist.

Section/Topic	#	Checklist Item	Reported on Page #
TITLE			
Title	1	Identify the report as a systematic review, meta-analysis, or both.	1
**ABSTRACT**			
Structured summary	2	Provide a structured summary including, as applicable: background; objectives; data sources; study eligibility criteria, participants and interventions; study appraisal and synthesis methods; results; limitations; conclusions and implications of key findings; systematic review registration number.	1
**INTRODUCTION**			
Rationale	3	Describe the rationale for the review in the context of what is already known.	2
Objectives	4	Provide an explicit statement of questions being addressed with reference to participants, interventions, comparisons, outcomes and study design (PICOS).	2
**METHODS**			
Protocol and registration	5	Indicate if a review protocol exists, if and where it can be accessed (e.g., Web address), and, if available, provide registration information including registration number.	N/A
Eligibility criteria	6	Specify study characteristics (e.g., PICOS, length of follow-up) and report characteristics (e.g., years considered, language, publication status) used as criteria for eligibility, giving rationale.	2
Information sources	7	Describe all information sources (e.g., databases with dates of coverage, contact with study authors to identify additional studies) in the search and date last searched.	3
Search	8	Present full electronic search strategy for at least one database, including any limits used, such that it could be repeated.	3
Study selection	9	State the process for selecting studies (i.e., screening, eligibility, included in systematic review, and, if applicable, included in the meta-analysis).	3
Data collection process	10	Describe method of data extraction from reports (e.g., piloted forms, independently, in duplicate) and any processes for obtaining and confirming data from investigators.	3
Data items	11	List and define all variables for which data were sought (e.g., PICOS, funding sources) and any assumptions and simplifications made.	3
Risk of bias in individual studies	12	Describe methods used for assessing risk of bias of individual studies (including specification of whether this was done at the study or outcome level), and how this information is to be used in any data synthesis.	3
Summary measures	13	State the principal summary measures (e.g., risk ratio, difference in means).	3–4
Synthesis of results	14	Describe the methods of handling data and combining results of studies, if done, including measures of consistency (e.g., I^2^) for each meta-analysis.	4
Risk of bias across studies	15	Specify any assessment of risk of bias that may affect the cumulative evidence (e.g., publication bias, selective reporting within studies).	4
Additional analyses	16	Describe methods of additional analyses (e.g., sensitivity or subgroup analyses, meta-regression), if done, indicating which were pre-specified.	4
**RESULTS**			
Study selection	17	Give numbers of studies screened, assessed for eligibility and included in the review, with reasons for exclusions at each stage, ideally with a flow diagram.	4–5
Study characteristics	18	For each study, present characteristics for which data were extracted (e.g., study size, PICOS, follow-up period) and provide the citations.	5–7
Risk of bias within studies	19	Present data on risk of bias of each study and, if available, any outcome level assessment (see item 12).	8
Results of individual studies	20	For all outcomes considered (benefits or harms), present, for each study: (a) simple summary data for each intervention group (b) effect estimates and confidence intervals, ideally with a forest plot.	8–14
Synthesis of results	21	Present results of each meta-analysis done, including confidence intervals and measures of consistency.	8–14
Risk of bias across studies	22	Present results of any assessment of risk of bias across studies (see Item 15).	8–14
Additional analysis	23	Give results of additional analyses, if done (e.g., sensitivity or subgroup analyses, meta-regression (see Item 16)).	8–14
**DISCUSSION**			
Summary of evidence	24	Summarize the main findings including the strength of evidence for each main outcome; consider their relevance to key groups (e.g., healthcare providers, users and policy makers).	14–17
Limitations	25	Discuss limitations at study and outcome level (e.g., risk of bias), and at review-level (e.g., incomplete retrieval of identified research, reporting bias).	16–17
Conclusions	26	Provide a general interpretation of the results in the context of other evidence, and implications for future research.	17
**FUNDING**			
Funding	27	Describe sources of funding for the systematic review and other support (e.g., supply of data); role of funders for the systematic review.	17

N/A, Not applicable.
