# Supplementary material for: Diet and Cardiovascular Disease Risk Among Individuals with Familial Hypercholesterolemia: Systematic Review and Meta-Analysis
Source: Nutrients. 2020 Aug 13;12(8):2436. doi: 10.3390/nu12082436 (PMC7468930; doi:10.3390/nu12082436)
Supplement: Supplementary file 1 [file nutrients-12-02436-s001.pdf]

Table S1. .Lipid profile of subjects assigned to low-fat/low-cholesterol diet and high-fat/high-cholesterol diet.

Variables/Trial	Last Visit Levels			
	High-Fat Diet		Low-Fat Diet	
	N	Mean (SD)	N	Mean (SD)
Chisholm 1994 [21]				
Total cholesterol, mmol/L	19	6.36 (0.98)	19	5.96 (0.75)
Triglycerides, mmol/L		1.49 (0.76)		1.55 (0.78)
High-density lipoprotein cholesterol, mmol/L		1.44 (0.38)		1.33 (0.35)
Low-density lipoprotein cholesterol, mmol/L		4.22 (0.93)		3.95 (0.70)
Very-low-density lipoprotein cholesterol, mmol/L		0.56 (0.41)		0.57 (0.37)

SD, standard deviation.

Table S2. Lipid profile of subjects assigned to omega-3 fatty acids and placebo.

Variables/Trial	Last Visit Levels			
	Omega-3 Fatty Acids Group		Placebo Group	
	N	Mean (SD)	N	Mean (SD)
Balestrieri 1996 [11]	14		14	
Total cholesterol, mmol/L		7.75 (1.27)		7.80 (1.14)
Triglycerides, mmol/L		1.02 (0.29)		1.26 (0.66)
High-density lipoprotein cholesterol, mmol/L		1.37 (0.54)		1.34 (0.47)
Low-density lipoprotein cholesterol, mmol/L		5.89 (1.29)		5.87 (1.34)
Apolipoprotein A-I, g/L		1.27 (0.47)		1.25 (0.42)
Apolipoprotein B100, g/L		2.04 (0.42)		2.05 (0.42)
Chan 2016 [20]	20		20	
Total cholesterol, mmol/L		4.20 (0.71)		4.58 (1.21)
Triglycerides, mmol/L		1.05 (0.40)		1.30 (0.63)
High-density lipoprotein cholesterol, mmol/L		1.12 (0.22)		1.19 (0.54)
Low-density lipoprotein cholesterol, mmol/L		2.54 (0.71)		2.81 (0.85)
Very-low-density lipoprotein cholesterol, mmol/L		0.57 (0.22)		0.77 (0.28)
Apolipoprotein B100, g/L		0.76 (0.13)		0.83 (0.27)
Lipoprotein (a), g/L		0.42 (0.45)		0.44 (0.49)
Hande 2019 [13]	34		34	
Total cholesterol, mmol/L		4.60 (0.80)		5.00 (1.10)
Triglycerides, mmol/L		0.84 (0.39)		1.15 (0.86)
High-density lipoprotein cholesterol, mmol/L		1.4 (0.4)		1.4 (0.4)
Low-density lipoprotein cholesterol, mmol/L		2.8 (0.9)		3.2 (0.9)

SD, standard deviation.

Table S3. Lipid profile of subjects assigned to low-fat diet regimes enriched with either monounsaturated fatty acids or polyunsaturated fatty acids.

Variables/Trial	Last Visit Levels			
	Monounsaturated Fat		Polyunsaturated Fat	
	N	Mean (SD)	N	Mean (SD)
Negele 2015 [19]	12		9	
Total cholesterol, mmol/L		5.55 (0.75)		6.28 (1.32)
Triglycerides, mmol/L		1.11 (0.63)		1.14 (0.54)
High-density lipoprotein cholesterol, mol/L		1.57 (0.24)		1.47 (0.39)
Low-density lipoprotein cholesterol, mol/L		3.46 (0.55)		4.30 (1.55)
Apolipoprotein A-I, g/L		1.40 (0.23)		1.41 (0.31)

Apolipoprotein B100, g/L	1.08 (0.32)	1.17 (0.30)

SD, standard deviation.

Table S4. Lipid profile of subjects assigned to plant stanols and placebo.

**Variables/Trial**	**Last Visit Levels**			
	**Plant Stanols Group**		**Placebo Group**	
	N	Mean (SD)	N	Mean (SD)
Gylling 1995 [16]	14		14	
Total cholesterol, mmol/L		6.81 (1.27)		7.62 (1.20)
Triglycerides, mmol/L		0.92 (0.45)		1.03 (0.49)
High-density lipoprotein cholesterol, mmol/L		1.25 (0.30)		1.20 (0.26)
Low-density lipoprotein cholesterol, mmol/L		4.65 (1.20)		5.47 (1.12)
Very-low-density lipoprotein cholesterol, mmol/L		0.25 (0.26)		0.26 (0.22)
Jakulj 2006 [14]	41		41	
Total cholesterol, mmol/L		6.47 (1.35)		7.00 (1.49)
Triglycerides, mmol/L		0.61 (0.24)		0.57 (0.31)
High-density lipoprotein cholesterol, mmol/L		1.35 (0.24)		1.38 (0.27)
Low-density lipoprotein cholesterol, mmol/L		4.77 (1.32)		5.24 (1.45)

SD, standard deviation.

Table S5. Lipid profile of subjects assigned to plant sterols and placebo.

**Variables/Trial**	**Last Visit Levels**			
	**Plant Sterols Group**		**Placebo Group**	
	N	Mean (SD)	N	Mean (SD)
Neil 2001 [15]	29		29	
Total cholesterol, mmol/L		6.84 (1.12)		7.20 (1.04)
Triglycerides, mmol/L		1.27 (0.65-3.80)		1.29 (0.66-3.93)
High-density lipoprotein cholesterol, mmol/L		1.49 (0.36)		1.43 (0.36)
Low-density lipoprotein cholesterol, mmol/L		4.65 (1.14)		4.99 (1.02)
Very-low-density lipoprotein cholesterol, mmol/L		0.73 (0.30)		0.81 (0.38)
Apolipoprotein A-I, g/L		1.41 (0.25)		1.47 (0.26)
Apolipoprotein B, g/L		1.46 (0.33)		1.47 (0.29)
Amundsen 2002 [10]	38		38	
Total cholesterol, mmol/L		6.87 (1.45)		7.48 (1.70)
Triglycerides, mmol/L		0.80 (0.37)		0.78 (0.33)
High-density lipoprotein cholesterol, mmol/L		1.26 (0.35)		1.25 (0.31)
Low-density lipoprotein cholesterol, mmol/L		5.25 (1.55)		5.88 (1.79)
Apolipoprotein A-I, g/L		1.32 (0.26)		1.35 (0.23)
Apolipoprotein B, g/L		1.32 (0.35)		1.48 (0.39)
De Jongh 2003 [12]	41		41	
Total cholesterol, mmol/L		6.27 (1.12)		7.06 (1.35)
Triglycerides, mmol/L		0.85 (0.36)		0.90 (0.40)
High-density lipoprotein cholesterol, mmol/L		1.31 (0.31)		1.29 (0.29)
Low-density lipoprotein cholesterol, mmol/L		4.58 (1.13)		5.40 (1.37)
Fuentes 2007 [22]	30		30	
Total cholesterol, mmol/L		5.74 (1.03)		5.84 (1.24)
Triglycerides, mmol/L		1.08 (0.61)		1.14 (0.49)
High-density lipoprotein cholesterol, mmol/L		1.39 (0.36)		1.37 (0.36)
Low-density lipoprotein cholesterol, mmol/L		3.83 (0.96)		3.96 (1.09)
Apolipoprotein A-I, g/L		1.46 (0.22)		1.47 (0.26)
Apolipoprotein B, g/L		1.11 (0.21)		1.14 (0.24)

SD, standard deviation.

Table S6. Lipid profile of subjects assigned to plant stanols and plant sterols.

**Variables/Trial**	**Last Visit Levels**			
	**Stanols Group**		**Sterols Group**	
	N	Mean (SD)	N	Mean (SD)
Ketomaki 2005 [23]	18		18	
Total cholesterol, mmol/L		5.65 (0.93)		5.71 (0.89)
Triglycerides, mmol/L		1.16 (0.51)		1.05 (0.38)
High-density lipoprotein cholesterol, mmol/L		1.32 (0.17)		1.37 (0.17)
Low-density lipoprotein cholesterol, mmol/L		3.81 (0.76)		3.86 (0.81)

SD, standard deviation.

Table S7. Lipid profile of subjects assigned to soy protein and control group.

**Variables/Trial**	**Last Visit Levels**			
	**Soy Group**		**Control Group**	
	N	Mean (SD)	N	Mean (SD)
Laurin 1991 [18]	9		9	
Total cholesterol, mmol/L		7.89 (1.02)		7.89 (1.02)
Triglycerides, mmol/L		0.80 (0.24)	`	1.02 (0.33)
High-density lipoprotein cholesterol, mmol/L		1.20 (0.21)		1.15 (0.18)
Low-density lipoprotein cholesterol, mmol/L		6.33 (1.02)		6.29 (1.11)
Very-low-density lipoprotein cholesterol, mmol/L		0.35 (0.12)		0.45 (0.15)
Apolipoprotein A-I, g/L		1.55 (0.09)		1.59 (0.15)
Apolipoprotein B, g/L		1.44 (0.06)		1.44 (0.18)
Helk 2019 [17]	13		13	
Total cholesterol, mmol/L		6.27 (0.96)		6.58 (1.03)
Triglycerides, mmol/L		0.81 (0.26)		0.9 (0.19)
High-density lipoprotein cholesterol, mmol/L		1.63 (0.26)		1.51 (12.6)
Low-density lipoprotein cholesterol, mmol/L		4.00 (0.78)		4.65 (1.08)
Very-low-density lipoprotein cholesterol, mmol/L		0.37 (0.12)		0.41 (0.09)
Apolipoprotein A-I, g/L		1.36 (0.13)		1.37 (0.15)
Apolipoprotein B, g/L		1.13 (0.19)		1.23 (0.21)
Lipoprotein (a), g/L		0.30 (0.29)		0.59 (0.59)

SD, standard deviation.

Table S8. Lipid profile of subjects assigned to increased and low protein intake.

**Variables/Trial**	**Last Visit Levels**			
	**High-Protein Group**		**Low-Protein Group**	
	N	Mean (SD)	N	Mean (SD)
Wolfe 1992 [26]	10		10	
Total cholesterol, mmol/L		5.7 (0.95)		6.1 (0.95)
Triglycerides, mmol/L		1.7 (0.32)		2.4 (0.95)
High-density lipoprotein cholesterol, mmol/L		0.97 (0.25)		0.89 (0.25)
Low-density lipoprotein cholesterol, mmol/L		4.5 (0.63)		4.8 (0.63)
Very-low-density lipoprotein cholesterol, mmol/L		0.49 (0.25)		0.66 (0.35)

SD, standard deviation.

Table S9. Lipid profile of subjects assigned to bezafibrate plus guar and bezafibrate alone.

**Variables/Trial**	**Last visit levels**			
	**Intervention group**		**Placebo group**	
	N	Mean (SD)	N	Mean (SD)
Wirth 1982 [24]	12		12	
Total cholesterol, mmol/L		8.52 (1.78)		9.09 (1.99)
Triglycerides, mmol/L		1.87 (0.49)		1.46 (0.79)
High-density lipoprotein cholesterol, mmol/L		1.24 (0.33)		1.42 (0.38)

Low-density lipoprotein cholesterol, mmol/L	6.08 (1.91)	7.91 (1.81)
Apolipoprotein A-I, g/L	1.21 (0.1)	1.17 (0.12)
Apolipoprotein B, g/L	1.55 (0.17)	2.05 (0.21)

SD, standard deviation.
